# Attack Rate, Case Fatality Rate and Predictors of Pertussis Outbreak During Pertussis Outbreak Investigation in Ethiopia: Systematic Review and Meta-Analysis

**DOI:** 10.1007/s44197-024-00234-4

**Published:** 2024-05-15

**Authors:** Mengistie Kassahun Tariku, Abebe Habtamu Belete, Daniel Tarekegn Worede, Simachew Animen Bante, Agumas Alemu Alehegn, Biniam Kebede Assen, Bantayehu Addis Tegegne, Sewnet Wongiel Misikir

**Affiliations:** 1 Department of Public Health, College of Health Science, Debre Markos University, Debre Markos, Ethiopia; 2 Department of Midwifery, College of Medicine and Health Science, Bahir Dar University, Bahir Dar, Ethiopia; 3 Amhara Regional State Public Health Institute, Bahir Dar, Ethiopia; 4 Department of Pharmacy, College of Health Science, Debre Markos University, Debre Markos, Ethiopia; 5 Department of Medical Laboratory Technology, Felege Hiwot Comprehensive Specialized Hospital, Bahir Dar, 680 Ethiopia

**Keywords:** Pertussis, Outbreak, Review, Ethiopia

## Abstract

**Background:**

Pertussis, a highly contagious, vaccine-preventable respiratory infection caused by Bordetella pertussis, is a leading global public health issue. Ethiopia is currently conducting multiple pertussis outbreak investigations, but there is a lack of comprehensive information on attack rate, case fatality rate, and infection predictors. This study aimed to measure attack rates, case fatality rates, and factors associated with pertussis outbreak.

**Methods:**

This study conducted a systematic review and meta-analysis of published and unpublished studies on pertussis outbreaks in Ethiopia from 2009 to 2023, using observational study designs, using the guideline Preferred Reporting Items for Systematic Reviews and Meta-Analyses (PRISMA). The study utilized databases like Science Direct, MEDLINE/PubMed, African Journals Online, Google Scholar and registers. The data were collected using an Excel Spreadsheet and then exported to STATA version 17 for analysis. Subgroup analysis was conducted to identify potential disparities. A random effects model was used to consider heterogeneity among studies. I^2^-squared test statistics were used to assess heterogeneity. The attack rate, case fatality rate, and odds ratio (OR) were presented using forest plots with a 95% confidence interval. Egger’s and Begg’s tests were used to evaluate the publication bias.

**Results:**

Seven pertussis outbreak investigations with a total of 2824 cases and 18 deaths were incorporated. The pooled attack and case fatality rates were 10.78 (95% CI: 8.1–13.5) per 1000 population and 0.8% (95% CI: 0.01–1.58%), respectively. The highest and lowest attack rates were in Oromia (5.57 per 1000 population and in the Amhara region (2.61 per 1000 population), respectively. Predictor of pertussis outbreak were being unvaccinated [odds ratio (OR) = 3.05, 95% CI: 1.83–4.27] and contact history [OR = 3.44, 95% CI: 1.69–5.19].

**Conclusion:**

Higher and notable variations in attack and case fatality rates were reported. Being unvaccinated and having contact history were the predictors of contracting pertussis disease in Ethiopia. Enhancing routine vaccination and contact tracing efforts should be strengthened.

## Introduction

A pertussis outbreak investigation involves identifying and confirming suspected outbreaks through prompt and intelligent use of appropriate procedures to contain the outbreak [1]. The aim of the pertussis outbreak investigation is to assess the outbreak’s magnitude, locate the source and population at risk, and initiate prompt case management in order to lower morbidity and mortality [2].

Pertussis (whooping cough), a highly communicable respiratory infection caused by Bordetella pertussis infection [3]. A cough of 14 days or more, or any duration with a paroxysm, or any duration cough with whoop is the suspect of pertussis case. A case that meets the clinical case definition and is linked epidemiologically or directly to a laboratory-confirmed case is known as a confirmed case [4, 5]. Catarrhal, paroxysmal, and convalescent are the three stages of pertussis. Grasping, fever, congestion, and sneezing are all part of the catarrhal stage. A severe cough, cyanosis, and rapid coughing are symptoms of the paroxysmal stage. Over the course of two to three weeks, the cough will lessen in intensity and eventually go away during the convalescent phase [1, 6, 7].

Pertussis can be transmitted through respiratory contact with infected individuals’ secretions, up to two weeks before and after symptoms appear [8, 9]. There are no known sources of pertussis in animals, insects, or vectors; humans are the only known reservoir for the disease [10, 11].

Globally, over 150,000 cases, with 160,700 children are dying annually. Africa has the highest global case and death rates of the disease, accounting for 33% of cases and 58% of deaths [12]. In 2016, Ethiopia reported 4,719 confirmed pertussis cases and 9 deaths [13].

Pertussis cases and outbreaks are primarily influenced by factors such as living in close proximity to an infected person, waning immunity post-vaccination, and not being immunized [10, 14]. Individuals without diphtheria, pertussis and tetanus (DPT) vaccines in the same household are 80–100% susceptible to exposure, while those immunized and living in the same household are 20% [15].

The highest likelihood of pertussis-related morbidity and mortality is present in infants and early childhood [16]. Young unvaccinated infants, under-vaccinated preschool children, and those under 6 months old are at higher risk for severe complications related to pertussis [17].

Vaccination is the most effective method for preventing pertussis in all age groups [18]. High vaccine coverage leads to high protection in children under five, while minor reductions can increase cases [1, 19]. Three doses of the pertussis vaccine, when completed, prevent 95% of deaths and 80% of cases. It has been demonstrated that incomplete vaccination can prevent severe morbidity; mortality is reduced by 50% and 80%, respectively, after one and two doses [1, 20].

Most African countries, except Morocco and Rwanda, have varying DPT3 coverage by 25%, with Ethiopia, Somalia, and Angola having low coverage and high dropout rates [21].

Ethiopia’s national vaccination strategy includes Pentavalent vaccines at 6, 10, and 14 weeks, but not pertussis booster. Despite good coverage, recent outbreaks of pertussis have been reported in various localities [22].

In 2009 and 2017, Ethiopia introduced advanced field epidemiology and frontline programs, respectively to improve outbreak identification and investigation [23]. Meta-analysis is a widely-used tool that integrates findings from various studies to inform decision-making in evidence-based medicine [24]. Ethiopia is currently conducting multiple pertussis outbreak investigations, but there is a lack of comprehensive information on attack rate, case fatality rate, and infection predictors. Therefore, this study aimed to measure the pooled attack rates, case fatality rates, and factors associated with pertussis outbreak.

## Methods

### Study Design and Searching Methods

A systematic review and meta-analysis of published or unpublished studies on pertussis/ whooping cough outbreaks were employed from December 1–25/2023 by using the guideline Preferred Reporting Items for Systematic Reviews and Meta-Analyses (PRISMA) in this study. The databases of Science Direct, MEDLINE/PubMed, African Journals Online, and Google Scholar were searched for published studies. The terms that were used were “Whooping cough or pertussis or Bordetella pertussis and attack rate or incidence and case fatality rate or mortality and determinants or risk factors and outbreak or epidemic and investigation or study or search and Ethiopia.”

### Study Selection and Eligible Criteria

All published or unpublished articles on pertussis/ whooping cough outbreaks investigations in Ethiopia were included in this systematic review and meta-analysis. The study designs used in the studies were observational study designs. The systematic review included studies on pertussis/ whooping cough outbreak investigations that were written, in English andaccessible online between 2009 and 2023. Outbreak investigations without attack rate or case fatality rate measurement were excluded.

### Measurement of Outcomes

The pertussis attack rate, which is determined by dividing the number of cases of the disease by the total number of population at risk and multiplied by 100, and the case fatality rate, which is determined by dividing the number of deaths from the disease by the total number of cases and multiplied by 100 [25], are the outcome variables. Additionally, factors influencing the likelihood of getting a Bordetella pertussis infection were outcome variables. The presence of the Bordetella pertussis infection was determined by contact and vaccination status. These factors were reported in odds ration with 95% CI.

### Quality Assessment and Data Extraction

Independently, two authors reviewed full-length articles, examined titles and abstracts, and evaluated the quality of studies to include or exclude. To ensure transparent communication and thorough analysis, the team convened with a third author in order to arrive at a consensus decision. The Joanna Briggs Institute quality check tool was used to assess each study’s quality [26]. The eight checklists in this quality check tool are designed to evaluate the following aspects of research quality: (1) evaluating inclusion and exclusion criteria; (2) summarizing the study subject and setting; (3) measuring outcome; (4) measuring exposure; (5) identifying confounding factors; (6) confounding factor control strategies; (7) suitable statistical analysis; and (8) objective and standard criteria applied. Seven studies that received a score of six out of eight were considered suitable for inclusion in the systematic review and meta-analysis.

Prior to collecting data, various kinds of literature were reviewed in order to adapt a standard tool. Two authors worked independently to develop the data extraction tool, and two more authors made revisions. The tool was approved by all authors prior to data collection. Author names, publication years, study designs, study periods, study settings, sample sizes, descriptive data analysis (attack rate, case fatality rate, outbreak duration, and vaccination status), and Pertussis/ Whooping cough outbreak factors were among the details included in the data extraction tool.

### Data Management and Analysis

To perform the meta-analysis, data was gathered, arranged, and imported into an Excel spreadsheet before being imported into STATA version 17. The thorough evaluation and consolidation of attack rates, case fatality rates, and variables influencing Bordetella pertussis infection was the primary objective of the systematic review and meta-analysis.

The attack rate, case fatality rate, and standard error data from each study were used to calculate the pooled attack and case fatality rates as well as the corresponding 95% confidence intervals (CI). The findings were visually represented using forest plots, which showed the odds ratios (OR) corresponding to variables linked to Bordetella pertussis infection as well as the 95% confidence intervals (CI) for attack and case fatality rates.

Subgroup analysis, which took into account variables like study design and geography, was done to look into any potential disparities. The meta-analysis used a random effects model to take into consideration the heterogeneity among the included studies.

The I^2^-squared test statistic and its associated p-value were used to assess the heterogeneity of the investigation. Heterogeneity was defined as a p-value less than 0.05. We used I^2^ 25, 50, and 75% statistics to indicate low, moderate, and high heterogeneity [27], respectively. A meta-analysis’s potential publication bias is often evaluated using Egger’s and Begg’s tests, with a p-value of less than 0.005 indicating the significance of the results [28].

## Results

### Study Selection

**Fig. 1:**
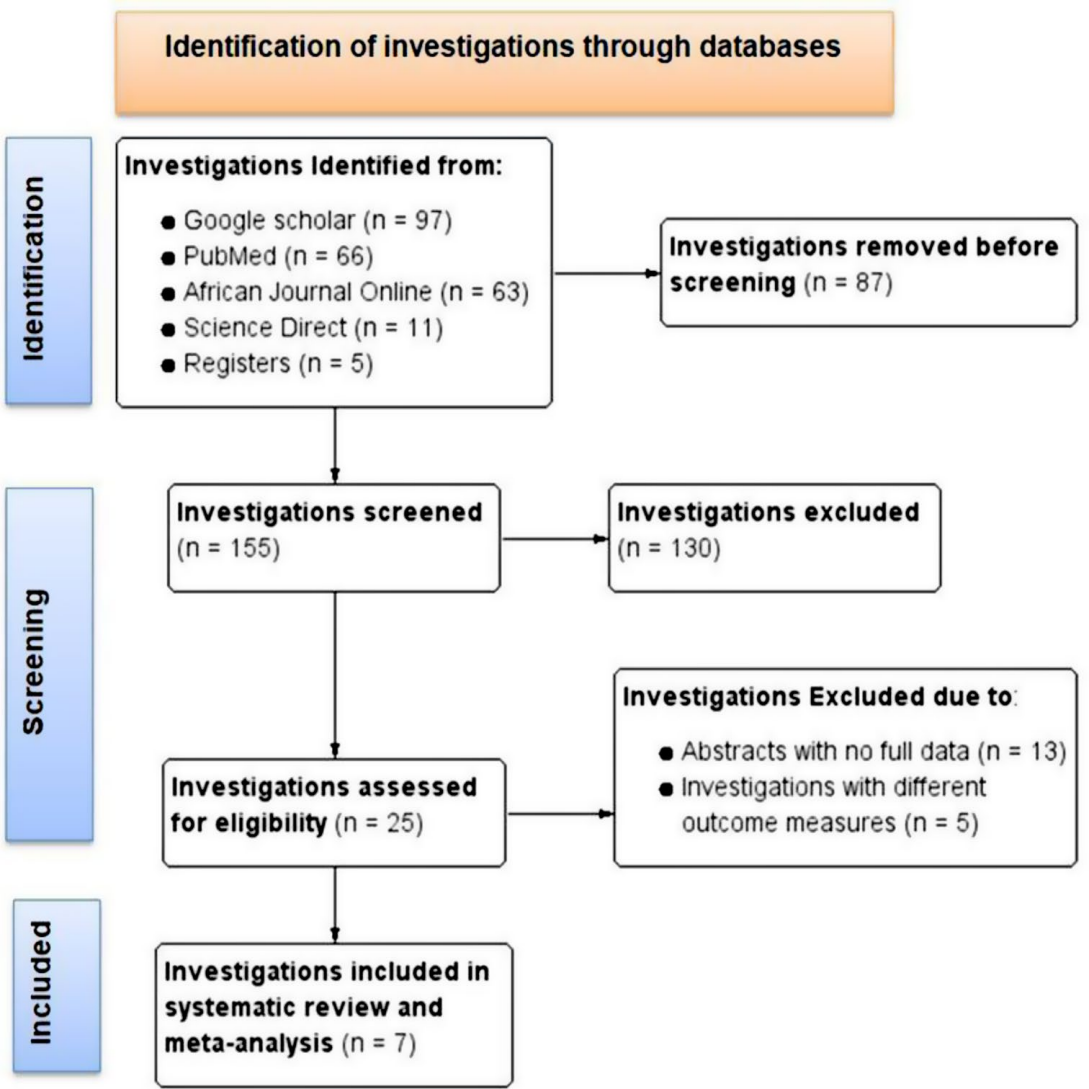
Flow diagram of pertussis outbreak investigation included in systematic review and meta-analysis in Ethiopia, 1980–2023

We were able to obtain a total of 242 records through electronic database searches. One hundred thirty investigations were removed from consideration after a preliminary screening process that evaluated titles, abstracts, and full article reviews. Following this, eligibility was assessed for 25 articles, of which 18 were rejected because of insufficient reporting. In the end, seven studies satisfied the requirements and were included in the systematic review and the meta-analysis (Fig. 1).

### Characteristics of Included Investigations

Seven different Pertussis/whooping cough outbreak investigations were included in this systematic review and meta-analysis, a total of 2824 confirmed cases of the disease and 18 deaths recorded (ages 1 month to 51 years) [4, 5, 29–33]. Of the seven studies, four had a case-control study design and the other three had a descriptive cross-sectional, study design. Five articles were conducted in the Amhara region [4, 5, 30, 32, 33], one in South Nation Nationality and People Region (SNNPR) [29], and one in Oromia region [31]. The included articles were investigated from 2017 to 2023, and the outbreak lasted from 21 days to 149 days (Table 1).

**Table 1:** Summary characteristics of studies included in a systematic review and meta-analysis of a pertussis outbreak investigation in Ethiopia, 2009–2023

Author	Study area	Study Period	Study design	Sample size	Outbreak duration	AR per 1000 Population	CFR (%)
Mitiku AD. et al., 2020	SNNPR	1st September 2018 to 9th January 2019	Cross-sectional	1840	149 days	17.080	0.33
Yeshanew A.D. et al., 2022	Amhara	March 27—April 30, 2019	Case-control	182	21 days	0.864	0
Almaw L. et al., 2019	Amhara	April 22-May 10, 2017	Case-control	120	60 days	1.352	0
Alamaw S.D. et al.	Amhara		Case-control	315	112 days	1.294	3.72
Badeso M.H. et al., 2022	Oromia	September 2018 to December 2018,	Cross-sectional	439	85	55.682	0.68
Wagaye et al., 2023	Amhara	December 3/2020 to January 05/2021	Cross-sectional	43	30	7.104	0
Mohammed S. et al., unpublished	Amhara	November 21–30,2022	Case-control	320	45	7.54	0.95

### Study bias Assessment Results

Every article was carefully evaluated, with studies receiving an 8 out of 8 being classified as good quality, and studies receiving a 6 or 7 being classified as medium risk. Using the aforementioned appraisal tools, no study was left out of the reviews. Cross-sectional and case-control studies were assessed using the following criteria (Table 2).

**Table 2:** Quality assessment checklist and result scores of each pertussis outbreak systematic review and meta-analysis in Ethiopia

Checklist	Articles						
	Mitiku AD. et al., 2020	Yeshanew A.D. et al., 2022	Almaw L. et al., 2019	Alamaw S.D. et al., 2017	Badeso M.H. et al., 2022	Wagaye et al., 2023	Mohammed S. et al., unpublished
Participation criteria were followed	✓	✓	✓	✓	✓	✓	✓
summarizing the study subject and setting	✓	✓	✓	✓	✓	✓	✓
objective and standard criteria applied	✓	✓	✓	✓	✓	✓	✓
measuring outcome	✓	✓	✓	✓	✓	✓	✓
measuring exposure	✓	✓	✓	✓	✓	✓	✓
identifying confounding factors	✓	✓	✓	✓	X	X	✓
confounding factor control strategies;	✓	✓	x	✓	X	X	✓
suitable statistical analysis	✓	✓	✓	✓	✓	✓	✓
Total score	8/8	8/8	7/8	8/8	6/8	6/8	8/8

### Attack Rate

**Fig. 2:**
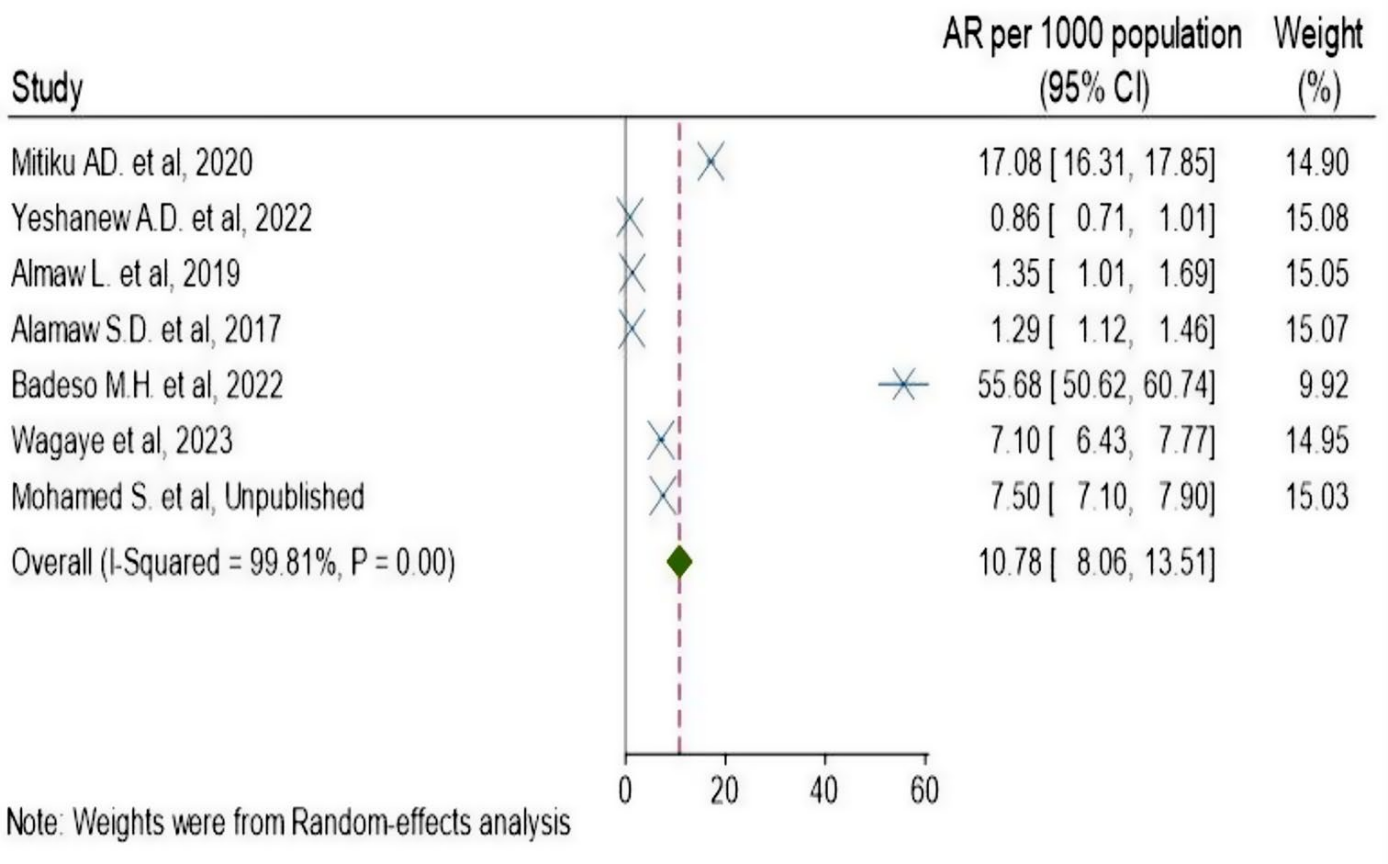
Forest plot of attack rate of pertussis outbreak in Ethiopia, 2009–2023

**Fig. 3:**
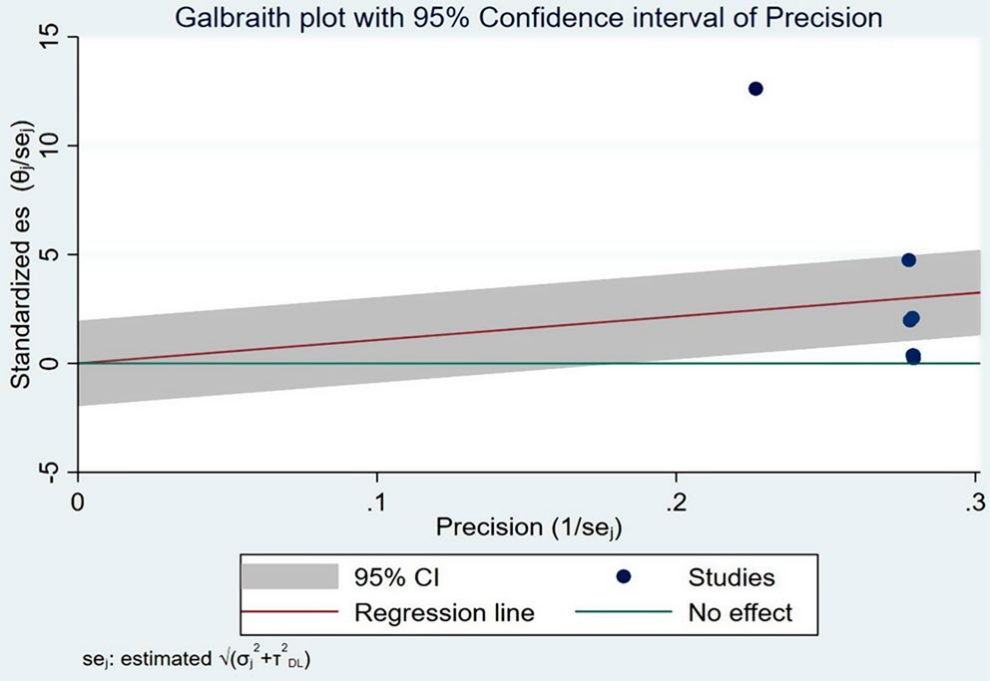
Galbraith plot with 95% CI of precision of Attack rate during pertussis outbreak investigations in Ethiopia, 2009–2023

**Fig. 4:**
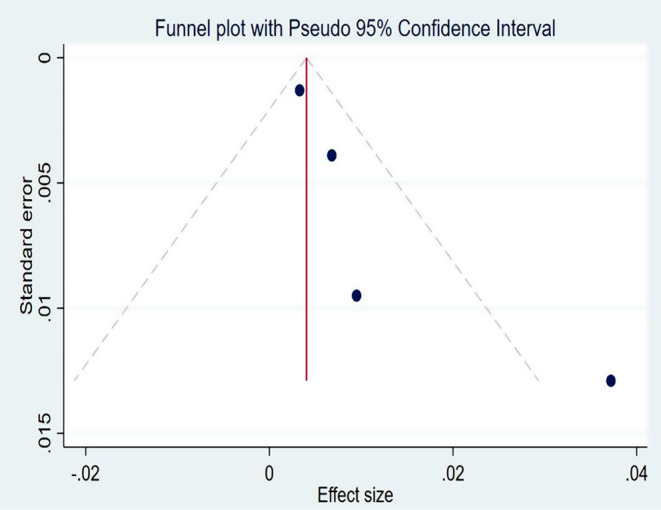
Funnel plot with Pseudo 95% CI to assess publication bias of the Meta-analysis of attack rate during pertussis outbreak investigations in Etiopia, 2009–2023

The systemic review and meta-analysis yielded a pooled attack rate (A.R.) of 10.78 (95% CI: 8.1–13.5) per 1000 population [4, 5, 30–34] (Fig. 2). The heterogeneity was significantly higher (p-value < 0.0001 and I^2^ = 99.81% (95% CI: 99.78-99.84%)) (Fig. 3). There was a noticeable publication bias found, with Eggers, *P* < 0.0001 and funnel plot (Fig. 4).

### Case Fatality Rate

**Fig. 5:**
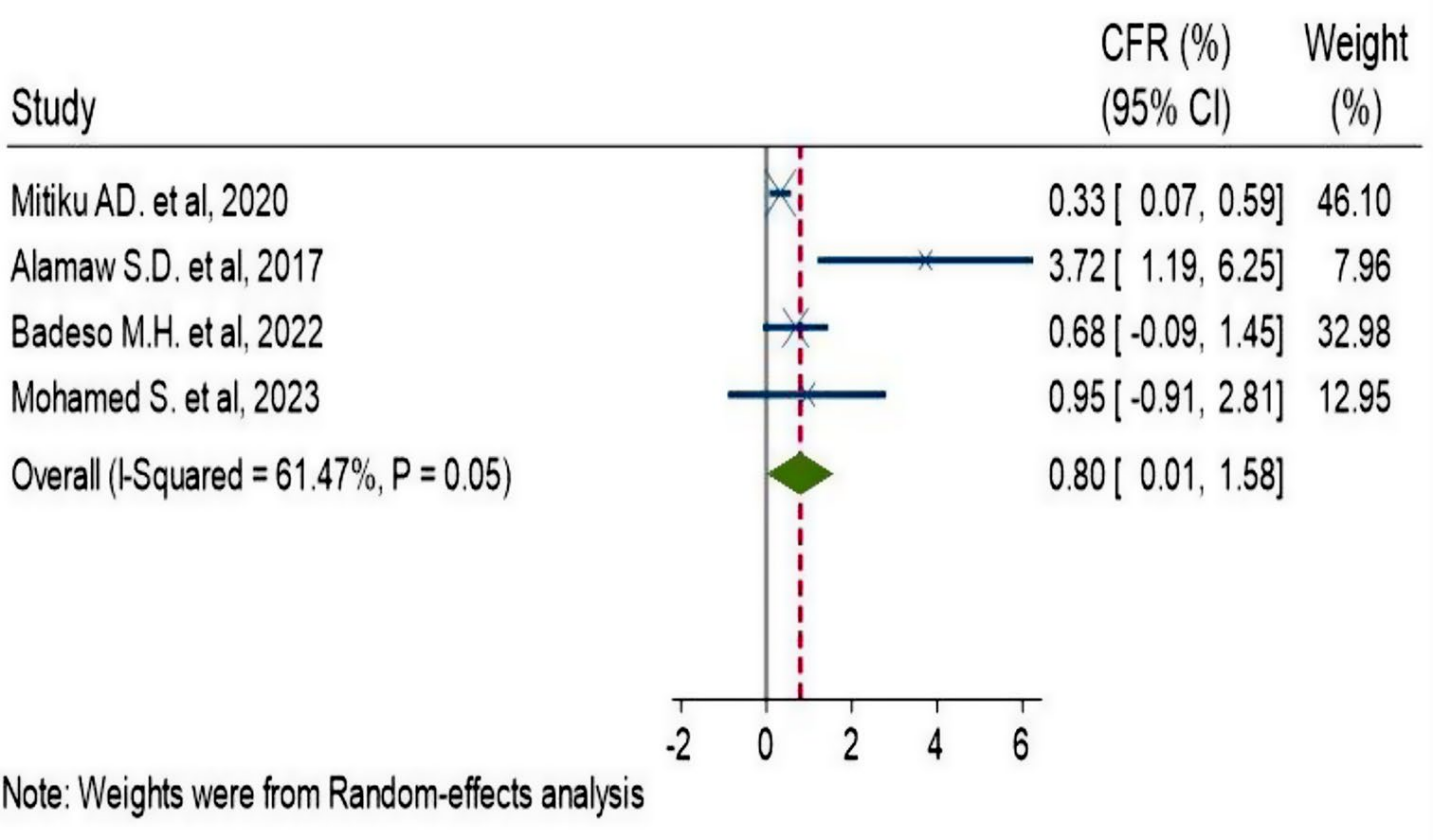
Forest plot of case fatality rate of pertussis outbreak in Ethiopia, 2009–2023

**Fig. 6:**
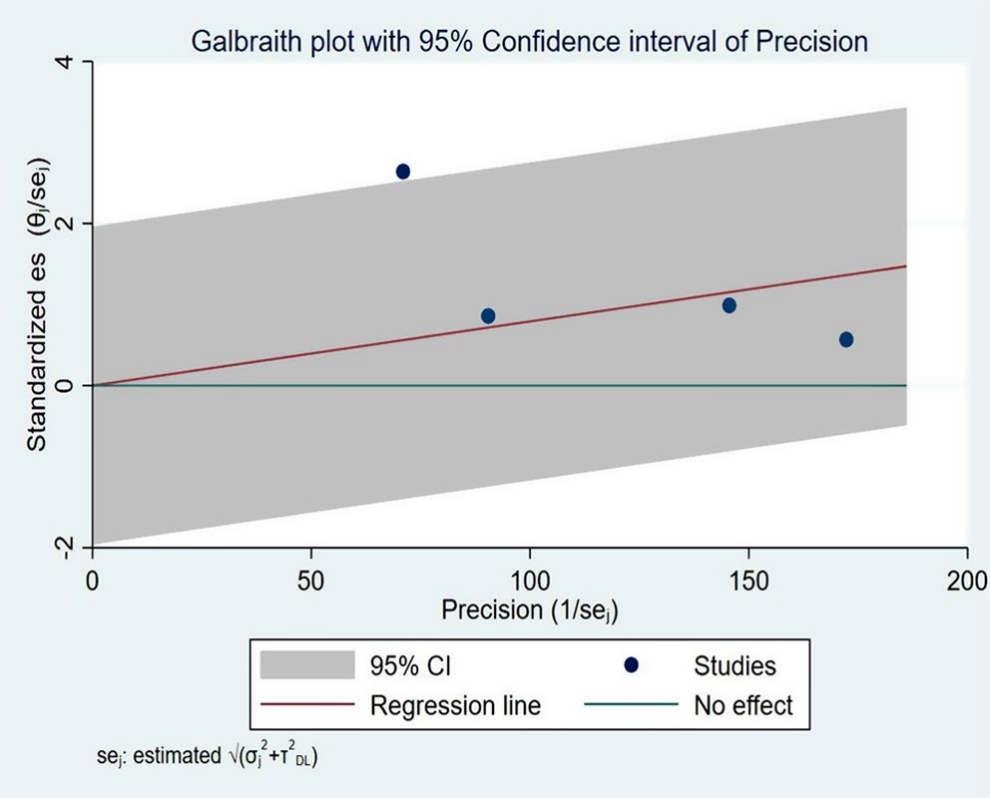
Galbraith plot with 95% CI of precision of case fatality rate during pertussis outbreak investigations in Ethiopia, 2009–2023

**Fig. 7:**
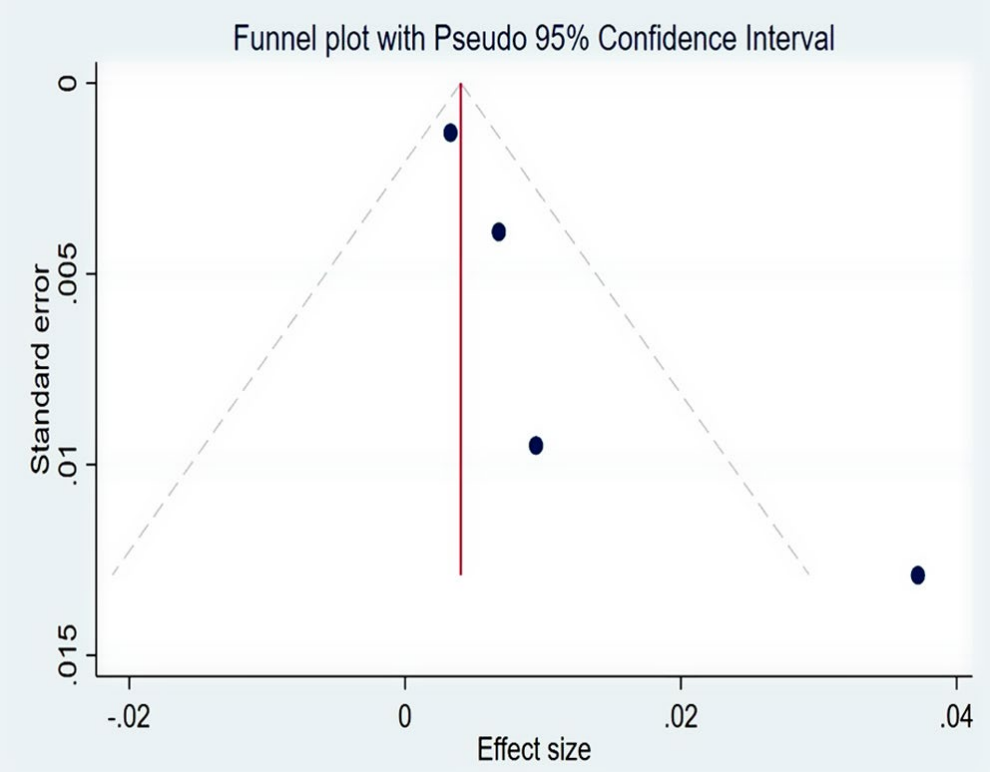
Funnel plot with Pseudo 95% CI to assess publication bias of the Meta-analysis of case fatality rate during pertussis outbreak investigations in Ethiopia, 2009–2023

The pooled CFR for this study was 0.8% (95% CI: 0.01–1.58%) [4, 28, 30, 31] (Fig. 5). Significantly moderate heterogeneity was present (p-value = 0.05 and I^2^ = 61.47% (95% CI: 0-87.1%)) (Fig. 6). There was a detectable publication bias with Egger tests, p-value = 0.015 and funnel plot (Fig. 7).

### Subgroup-Analysis

Based on a subgroup analysis of attack rates by region, the Oromia region had the highest attack rate, with 55.7 (95% CI: 50.6–60.7) per 1000 population. The Amhara region had the lowest reported AR, 2.61 (95% CI: 1.81–3.40) per 1000 population. A higher attack rate was reported in the cross-sectional study design: 26.30 (95% CI: 11.35–41.26) per 1000 population (Table 3).

**Table 3:** Subgroup analysis for an attack rate of pertussis outbreak in Ethiopia, 2009–2023

Subgroup	Number of studies	Sample size	AR per 1000(95% CI)	I^2^ (%)	*P*-value
*By region*					
Amhara	5	980	2.61 (1.81–3.40)	96.77	0.0001
Oromia	1	439	55.7 (50.6–60.7)	-	-
SNNPR	1	1840	17.1 (16.3–17.9)	-	-
*By study Design*					
Case-control	4	617	2.05 (1.32–2.78)	96.78	0.0001
Cross-sectional	3	2322	26.30 (11.35–41.26)	99.35	0.0001

### Predictor of Pertussis Infection

Three investigations into outbreaks found a significant association between the vaccination status—vaccinated or unvaccinated—and the likelihood of contracting pertussis disease [4, 5]. According to the meta-analysis, individuals who had not received the pertussis vaccination had an almost threefold increased risk of getting the illness (OR = 3.05, 95% CI: 1.83–4.27). The evidence of heterogeneity (I^2^ = 0, P-value = 0.6623) demonstrated the absence of heterogeneity.

Contact history was found to be a significant risk factor for contracting pertussis in three outbreak investigations [4, 5, 30]. The individuals who had contact history were more than 3 times more likely in contracting pertussis disease (OR = 3.44, 95% CI: 1.69–5.19). The heterogeneity test showed that there was no heterogeneity (I^2^ = 0, P-value = 0.6250) (Table 4).

**Table 4:** Predictors of pertussis infection during outbreak investigation in Ethiopia, 2009–2023

Variables	N*o* studies	Sample size	OR(95% CI)	I^2^ (%)	*P*-value
*Vaccinated*					
Yes	3	817	3.05 (1.83–4.27)	0	0.6623
No					
*Contact*					
Yes	4	937	3.44 (1.69–5.19)	0	0.6250
No					
*Window*					
Yes	2	302	2.76 (0.72–4.81)	0	0.8479
No					

## Discussion

In order to assess the total evidence of the pertussis outbreak investigation in Ethiopia, a systematic review and meta-analysis were carried out. During the course of the outbreak investigation, the pooled attack rate of 10.78 (95% CI: 8.06–13.51) per 1000 has been estimated. The highest and lowest attack rates were 55.682 per 1000 population [31] and 0.864 per 1000 population [5], respectively. There could be several reasons for this variation in attack rate between outbreaks, including the population’s vaccination status, the duration of the outbreak, its range, and at the time the intervention was launched. Differences may also exist in the response-to-action threshold and the duration of the outbreak investigation.

In four studies, the age group of children under five years old had the highest age-specific attack rate, 197.7/1000 [31], 73.6/1000 [30], 6.8/1000 [4], and 5.5/1000 (929) populations. The age group of 5 to 9 years old had the highest attack rate (245 per 1000 population) according to one outbreak investigation [5]. Children under the age of four were the most affected group population in another investigation [33].

A sex-specific attack rate was found to be similar in four outbreak investigations [5, 30–32], whereas the highest attack rate was found in females in two outbreak investigations [4, 33].

Six articles [4, 5, 29, 31–33] used the epidemic curve to plot the outbreak over time, while the epidemic curve was not used to characterize the outbreak in terms of time in one outbreak investigation [30]. Between September and January, three distinct outbreaks had occurred [29, 33].

Two outbreak investigations used place specific attack rate but did not use map [5, 31], while three outbreak investigations did not use place specific attack rate [4, 30, 33]. In one outbreak investigation, place specific attack rate and map were utilized [29]. The attack rate showed significant regional variation. Between 0.864 and 7.5 attacks per 1000 population were noted in the Amhara region [4, 5, 30, 32, 33], 55.682 attacks per 1000 population in the Oromia region [31], and 17.080 attacks per 1000 population in the SNNPR region [29]. According to subgroup analysis, the Amhara region had the lowest pooled attack rate (2.61 per 1000 population) [4, 5, 30, 32, 33], while the Oromia region had the highest pooled attack rate (55.682 per 1000 population) [31]. This discrepancy could result from a difference in the timing of the outbreak response’s start and detection. After the outbreak had been ongoing for two weeks and two months, respectively, the district health office and the zonal health department in the Oromia region responded to it [31]. Another explanation for this variation could be the different denominator used to calculate the attack rate. In the Oromia region, the smallest administrative unit, the affected Keble [31], served as the denominator, whereas in other outbreak investigations, the district population—which included people living outside of affected Kebeles—was used. This could inflate the AR in the Oromia region.

A higher attack rate—26.30 (95% CI; 11.35–41.26) per 1000 population—was found in subgroup analysis of a cross-sectional study design. This could be as a result of the case-control study design’s primary focus on risk factors identification during data collection, which could jeopardize active case search.

In this study, the pooled case fatality rate was 0.8% (95% CI: 0.01–1.58%). The case fatality rate in four outbreaks [4, 29, 31, 32] ranged from 0.33 to 3.72%, while in three other outbreak investigations, the CFR was zero [5, 30, 33]. The variation may be attributed to disparities in the duration of the outbreak. The Amhara region experienced an outbreak with a CFR of 3.72% for 112 days [4], the Oromia region experienced an outbreak with a CFR of 0.68% for 85 days [31] and the SNNPR experienced an outbreak with a CFR of 0.33% for 149 days [29]. The duration of outbreak of the remaining outbreak investigations, which had zero CFR, was 60 days [30], 30 days [33] and 21 days [5]. A discrepancy in the intervention’s start time could be another factor. Three fatalities were recorded in the Oromia region prior to the start of the outbreak control and public health response measures. Additional deaths might have been avoided if clinical case management had started sooner [31].

In two outbreak investigation, the highest CFR was observed at the age of 5–9 years, 6.3% [4] and 1.4% [31]. The age group ≤ 5 years had the highest CFR, 0.87% [29] in another outbreak investigation. In one unpublished outbreak investigation, the highest CFR was reported at the age group of < 1 year, 17% [32]. This variation might be due to difference in immunization status across age group.

In three outbreak investigation, females had the highest CFR, 2% [32], 0.91% [31] and 4.34% [4].

Childhood pertussis vaccination provides limited protection, but when completed, it prevents 95% of deaths and 80% of cases with three doses [1, 19]. Individuals without diphtheria, pertussis and tetanus (DPT) vaccines in the same household are 80–100% susceptible to exposure [13]. The study found that individuals who did not receive the pertussis vaccination had a nearly threefold increased risk of contracting the disease [4, 5, 32]. Two outbreak investigations revealed that all cases had unknown vaccination status [32, 34], while in two other investigations, 41% of cases completed the DPT 3 dose [5, 30]. A study in Janamora district, Amhara region revealed 86.6% of cases were unvaccinated [30], while 51.2% completed DPT3 in Mekdela district, South Wollo zone, Amhara region [4]. Another study in Mahal Saynt district, South Wollo Zone, Amhara Region showed that 34.29% of cases were not vaccinated [32]. The article in the Oromia region, the study setting, had 100% official reported vaccination coverage [31]. In four outbreak investigation, there was no regular routine immunization service and kebeles health posts didn’t have functional refrigerators for the storage of vaccines [4, 30, 31, 33], the study in SNNPR showed that investigation team did not find continuously recorded temperature monitoring tools [29].

Pertussis cases and outbreaks are primarily influenced by factors such as living in close proximity to an infected person [10, 13]. The individuals who had contact history were more than three times more likely in contracting pertussis disease as compared to individuals who had no contact history. This is congruent with the study conducted in Australia [34].

Even though this study provided the consolidated evidences for pertussis outbreak investigation in Ethiopia, there are some limitations. First, this study only included pertussis outbreak investigations, which were written in English and accessible online. This might under or overestimate attack rate and case fatality rate. Secondly, the cross-sectional and case-control study designs employed in all of the included outbreak investigations restrict the ability to evaluate causal relationships.

## Conclusion

Our results showed that higher as compared with 2023 Provisional Pertussis Surveillance Report and significant differences in attack and case fatality rates between the various study regions. In Ethiopia, the risk factors for catching pertussis were not getting vaccinated and having a history of contact. The overbearing measures include enhancing routine vaccination and contact tracing efforts should be strengthened.

## Data Availability

The data sets generated during the current study are available from the corresponding author upon reasonable request.

## Abbreviations

AR: Attack rate
CFR: Case fatality rate
CI: Confidence interval
DPT: diphtheria pertussis tetanus and OR: Odds ratio

## References

[1] Blain A, Tiwari T. Manual for the Surveillance of Vaccine-Preventable Diseases. Atlanta, GA: US Department of Health and Human Services. 2017;500:2017.

[2] Forsyth K, Tan T, von König C-HW, Caro JJ, Plotkin S (2005). Potential strategies to reduce the burden of pertussis. Pediatr Infect Dis J.

[3] Trainor EA, Nicholson TL, Merkel TJ. Bordetella pertussis transmission. Pathogens Disease. 2015;73(8). 10.1093/femspd/ftv068 PMC4626651 26374235

[4] Alamaw SD, Kassa AW, Gelaw YA (2017). Pertussis outbreak investigation of Mekdela district, south Wollo Zone, Amhara region, north-west Ethiopia. BMC Res Notes.

[5] Yeshanew AG, Lankir D, Wondimu J, Solomon S (2022). Pertussis outbreak investigation in Northwest Ethiopia: a community based study. PLoS ONE.

[6] Kline JM, Lewis WD, Smith EA, Tracy LR, Moerschel SK (2013). Pertussis: a reemerging infection. Am Family Phys.

[7] Organization WH. WHO vaccine-preventable diseases: monitoring system: 2009 global summary. World Health Organization; 2009.

[8] Control, CfD. Prevention. Manual for the surveillance of vaccine-preventable diseases. Atlanta: Centers for Disease Control and Prevention, 1997. 2003.

[9] Gopal DP, Barber J, Toeg D (2019). Pertussis (whooping cough). BMJ.

[10] Hamborsky J, Kroger A (2015). Epidemiology and prevention of vaccine-preventable diseases.

[11] Ryu S, Kim JJ, Chen M-Y, Jin H, Lee HK, Chun BC (2018). Outbreak investigation of pertussis in an elementary school: a case-control study among vaccinated students. Clin Experimental Vaccine Res.

[12] Yeung KHT, Duclos P, Nelson EAS, Hutubessy RCW (2017). An update of the global burden of pertussis in children younger than 5 years: a modelling study. Lancet Infect Dis.

[13] Taye S, Tessema B, Gelaw B, Moges F. Assessment of pertussis vaccine protective effectiveness in children in the Amhara regional state, Ethiopia. International journal of microbiology. 2020;2020. 10.1155/2020/8845835 PMC7579676 33110430

[14] Wensley A, Hughes G, Campbell H, Amirthalingam G, Andrews N, Young N (2017). Risk factors for pertussis in adults and teenagers in England. Epidemiol Infect.

[15] Hughes MM, Englund JA, Kuypers J, Tielsch JM, Khatry SK, Shrestha L, et al. Population-based pertussis incidence and risk factors in infants less than 6 months in Nepal. J Pediatr Infect Dis Soc. 2017;6(1):33–9. 10.1093/jpids/piw079 PMC5907881 28073985

[16] Calvert A, Karampelas K, Andrews N, England A, Hallis B, Jones CE et al. Optimising the timing of whooping cough immunisation in MUMs (OpTIMUM): a randomised controlled trial investigating the timing of pertussis vaccination in pregnancy. Lancet Microbe. 2021. 10.1016/S2666-5247(22)00332-9 37080224

[17] Baxter R, Bartlett J, Rowhani-Rahbar A, Fireman B, Klein NP. Effectiveness of pertussis vaccines for adolescents and adults: case-control study. BMJ. 2013;347. 10.1136/bmj.f4249 23873919

[18] Powell-Jackson T, Fabbri C, Dutt V, Tougher S, Singh K (2018). Effect and cost-effectiveness of educating mothers about childhood DPT vaccination on immunisation uptake, knowledge, and perceptions in Uttar Pradesh, India: a randomised controlled trial. PLoS Med.

[19] Crowcroft N, Stein C, Duclos P, Birmingham M (2003). How best to estimate the global burden of pertussis?. Lancet Infect Dis.

[20] Mosser JF, Gagne-Maynard W, Rao PC, Osgood-Zimmerman A, Fullman N, Graetz N (2019). Mapping diphtheria-pertussis-tetanus vaccine coverage in Africa, 2000–2016: a spatial and temporal modelling study. Lancet.

[21] Wolter N, Cohen C, Tempia S, Walaza S, Moosa F, du Plessis M (2021). Epidemiology of Pertussis in individuals of all ages hospitalized with respiratory illness in South Africa, January 2013—December 2018. Clin Infect Dis.

[22] Argaw MD, Desta BF, Tsegaye ZT, Mitiku AD, Atsa AA, Tefera BB (2022). Immunization data quality and decision making in pertussis outbreak management in southern Ethiopia: a cross sectional study. Archives Public Health.

[23] Kebebew T, Woldetsadik MA, Barker J, Cui A, Abedi AA, Sugerman DE (2023). Evaluation of Ethiopia’s field epidemiology training program–frontline: perspectives of implementing partners. BMC Health Serv Res.

[24] Bello A, Wiebe N, Garg A, Tonelli M. Evidence-based decision-making 2: systematic reviews and meta-analysis. Clin Epidemiology: Pract Methods. 2015:397–416. 10.1007/978-1-4939-2428-8_24 25694324

[25] Muller A, Leeuwenburg J, Pratt D (1986). Pertussis: epidemiology and control. Bull World Health Organ.

[26] Porritt K, Gomersall J, Lockwood C (2014). JBI’s systematic reviews: study selection and critical appraisal. AJN Am J Nurs.

[27] Sedgwick P. Meta-analyses: what is heterogeneity? Bmj. 2015;350. 10.1136/bmj.h1435 25778910

[28] Furuya-Kanamori L, Xu C, Lin L, Doan T, Chu H, Thalib L (2020). P value–driven methods were underpowered to detect publication bias: analysis of Cochrane review meta-analyses. J Clin Epidemiol.

[29] Mitiku AD, Argaw MD, Desta BF, Tsegaye ZT, Atsa AA, Tefera BB (2020). Pertussis outbreak in southern Ethiopia: challenges of detection, management, and response. BMC Public Health.

[30] Almaw L, Bizuneh H. Pertussis outbreak investigation in Janamora district, Amhara Regional State, Ethiopia: a case-control study. Pan Afr Med J. 2019;34(1). 10.11604/pamj.2019.34.65.19612 PMC6859057 31762929

[31] Badeso MH, Kalili FS, Bogale NB. Pertussis outbreak investigation in Likimsa-Bokore kebele, Meda Walebu district, Bale zone, Oromia region, Ethiopia, 2019. 2021. 10.11604/pamj.2024.48.37.20269 PMC11399468 39280818

[32] Seid Mohammed ZA, Pertussis outbreak investigation in Mahal Saynt District, South Wollo Zone. Ethiopia: Amhara Region; 2023.

[33] Wagaye FE, Asrat A, Shimekaw B, Hassen M, Terefe W, Gelaw A (2023). Pertussis outbreak investigation in south Gondar zone, Northwest, Ethiopia. Public Health.

[34] Kovitwanichkanont T. Public health measures for pertussis prevention and control. Aust N Z J Public Health. 2017;41(6). 10.1111/1753-6405.12732 29044817

